# Aberrant Epstein-Barr virus antibody patterns and chronic lymphocytic leukemia in a Spanish multicentric case-control study

**DOI:** 10.1186/1750-9378-10-5

**Published:** 2015-02-09

**Authors:** Delphine Casabonne, Yolanda Benavente, Claudia Robles, Laura Costas, Esther Alonso, Eva Gonzalez-Barca, Adonina Tardón, Trinidad Dierssen-Sotos, Eva Gimeno Vázquez, Marta Aymerich, Elias Campo, Gemma Castaño-Vinyals, Nuria Aragones, Marina Pollan, Manolis Kogevinas, Hedy Juwana, Jaap Middeldorp, Silvia de Sanjose

**Affiliations:** Unit of Infections and Cancer (UNIC), IDIBELL, Institut Català d’Oncologia, L’Hospitalet de Llobregat, Av. Gran Via 199 - 203, 2°; 08908 L’Hospitalet de Llobregat, Barcelona, Spain; CIBER Epidemiología y Salud Pública (CIBERESP), Madrid, Spain; Department of Pathology, Hospital Universitari de Bellvitge, L’Hospitalet de LLobregat, Barcelona, Spain; Hematology, L’ Hospitalet de Llobregat, IDIBELL, Institut Català d’ Oncologia, Barcelona, Spain; Faculty of Medicine, University of Oviedo, Oviedo, Asturias; Faculty of Medicine, University of Cantabria- IDIVAL, Santander, Spain; Hematology, Hospital del Mar, Barcelona, Spain; Hematopathology Unit, Pathology Department, Hospital Clínic and University of Barcelona, Institute of Biomedical Research August Pi i Sunyer (IDIBAPS), Barcelona, Spain; Centre for Research in Environmental Epidemiology (CREAL), Barcelona, Spain; Hospital del Mar Medical Research Institute (IMIM), Barcelona, Spain; Universitat Pompeu Fabra (UPF), Barcelona, Spain; National Center for Epidemiology, Carlos III Institute of Health, Madrid, Spain; Instituto de Investigación Sanitaria (IIS) of Hierro, Majadahonda, Spain; National School of Public Health, Athens, Greece; Department Pathology, VU University medical center, Amsterdam, The Netherlands

**Keywords:** Chronic lymphocytic leukemia, Epstein-Barr virus, Serology, Case-control, Smoking

## Abstract

**Background:**

Epstein-Barr virus (EBV)-related malignancies harbour distinct serological responses to EBV antigens. We hypothesized that EBV serological patterns can be useful to identify different stages of chronic lymphocytic leukemia.

**Methods:**

Information on 150 cases with chronic lymphocytic leukemia and 157 frequency-matched (by age, sex and region) population-based controls from a Spanish multicentre case-control study was obtained. EBV immunoglobulin G serostatus was evaluated through a peptide-based ELISA and further by immunoblot analysis to EBV early antigens (EA), nuclear antigen (EBNA1), VCA-p18, VCA-p40 and Zebra. Two independent individuals categorized the serological patterns of the western blot analysis. Patients with very high response and diversity in EBV-specific polypeptides, in particular with clear responses to EA-associated proteins, were categorized as having an abnormal reactive pattern (ab_EBV). Adjusted odds ratios (OR) and 95% confidence interval (CI) were estimated using logistic regression models.

**Results:**

Almost all subjects were EBV-IgG positive (>95% of cases and controls) whereas ab_EBV patterns were detected in 23% of cases (N = 34) and 11% of controls (N = 17; OR: 2.44, 95% CI, 1.29 to 4.62; P = 0.006), particularly in intermediate/high risk patients. Although based on small numbers, the association was modified by smoking with a gradual reduction of ab_EBV-related OR for all Rai stages from never smokers to current smokers.

**Conclusions:**

Highly distinct EBV antibody diversity patterns revealed by immunoblot analysis were detected in cases compared to controls, detectable at very early stages of the disease and particularly among non smokers. This study provides further evidence of an abnormal immunological response against EBV in patients with chronic lymphocytic leukemia.

**Electronic supplementary material:**

The online version of this article (doi:10.1186/1750-9378-10-5) contains supplementary material, which is available to authorized users.

## Introduction

The gamma-herpes Epstein-Barr virus (EBV) infects and persists in human B-cells by exploiting the B-cells environment to maintain its life cycle and by avoiding the host’s immune surveillance with limited expression of viral proteins [1]. Healthy EBV carriers display anti-EBV antibodies to only a limited number of EBV proteins, including Esptein-Barr nuclear antigen 1 (EBNA1), viral capsid antigen (VCA)-p18, VCA-p40 (BdRF1) and Zebra (BZLF1) whereas a small proportion of healthy carriers with subclinical virus reactivation produces antibodies to early antigen (EA) [1]. Contrary, high anti-VCA and anti-early antigen-diffuse (EAd) titers have been observed in EBV related malignancies such as Hodgkin lymphoma and nasopharyngeal carcinoma [2] but the role of EBV in chronic lymphocytic leukemia (CLL) remains unclear [3–6].

Despite CLL malignant cells being generally EBV negative, EBV has been proposed to play an indirect role in the genesis or progression of CLL [5, 7, 8]. Data on EBV serological biomarkers is sparse [3–6]. Using data from the European case-control study EpiLymph, de Sanjose *et al* found that CLL patients were 3 times more likely to have an aberrant EBV antibody pattern (ab_EBV), mainly reflected by excessively high EA response, than controls, while no association with other lymphoma subtypes was observed [5]. Patients with ab_EBV were characterized by a more diverse pattern of antibody reactivity, yielding a broadly reactive immunoblot profile. In a nested case-control study within the Physicians’ and Nurses’ Health Studies, CLL patients showed a pattern also suggestive of an aberrant viral replication indicated by elevated anti-EBNA2 and anti-VCA and a EBNA1/EBNA2 ratio less than or equal to 1 compared to controls [3]. Similarly, de Roos *et al* examined the prospective antibody response to anti-VCA, EBNA1, EAd using multiplex technology and EBV DNA load samples collected before diagnosis in 142 CLL/prolymphocytic leukemia patients and their matched controls [4]. A lower EBNA1 response with high levels of both EBV DNA and anti-EAd antibodies were associated with an increased risk of CLL [4]. In a recent prospective study increased EAd and Zebra antibodies were observed in CLL cases although based on a limited series [6]. Mental and medical (such as use of corticosteroids) “stressors” have been strongly implicated in the reactivation from the EBV latent stage to a lytic stage [9–11] and steroids are used to control nausea or as part of some CLL treatments. Smoking as also being associated with EBV seropositivity and reactivation of EBV [12] but not with CLL [13–15]. Here, we hypothesized that EBV serological patterns differ by different stages of chronic lymphocytic leukemia. Using data from the Spanish CLL multicentric case-control (MCC-Spain), the present work looked at the serological patterns to EBV and its association with Rai stages and potential effect modifications from epidemiological questionnaire in CLL cases and their respective controls.

## Materials and methods

### The Spanish multicase-control study (MCC-Spain study, http://www.mccspain.org)

Cases were recruited within the MCC-Spain study and in collaboration with the International Cancer Genome Consortium on Chronic Lymphocytic Leukemia Project *(ICGC-CLL,*http://www.cllgenome.es*and*http://www.icgc.org). The main objective of the MCC-Spain study is to investigate lifetime environmental, infectious, medical and occupational exposures, and genetic factors associated with 5 cancer sites. In brief, the recruitment took place between March 2010 and July 2012 and includes pathological confirmed cases of CLL enrolled in 11 hospitals of 5 Spanish areas (Asturias, Barcelona, Cantabria, Girona and Granada), together with a common set of frequency-matched population controls, randomly selected from lists of primary care centres. Response rate for CLL was 87% and for controls with valid phone number response rates were 48%, 58% and 60% for Asturias, Barcelona and Cantabria, respectively. Information was requested through a computerized face-to-face interview (the questionnaire is available at http://www.mccspain.org). A signed consent form was requested for acceptance of analysis of biological material and verification of clinical information. Ethical approval was granted for each participating centre of the study.

### Study participants

We calculated that a sample size of 150 case-control pairs would be required to detect a minimum odds ratio of 3.0 [5] with alpha level 0.05 and 95% statistical power. Finally, 150 cases and 157 controls were enrolled in the present study (7 EBV-tested cases were dropped due to invalid diagnosis). Both newly diagnosed and prevalent cases were included in the study. Given the indolent course of the disease, incident cases were defined as newly diagnosed patients with CLL that have been recruited within 1 year of diagnosis and the remainders were classified as prevalent cases.

### Outcome definition

CLL cases were diagnosed according to the criteria of the International Workshop on Chronic Lymphocytic Leukemia [16]. All diagnoses were morphologically and immunologically confirmed using flow cytometry immunophenotype and complete blood count. Disease severity was evaluated using the Rai staging system obtained at the time of interview from medical records and verified by local haematologists. For this study, Rai stages were categorised into two groups: A) low risk category including asymptomatic patients with lymphocytosis only (Rai 0) and B) intermediate/high risk category including patients with lymphocytosis with or without lymphadenopathy, hepatomegaly, splenomegaly, anemia and/or thrombocytopenia (Rai I-IV). CLL and small lymphocytic lymphoma (SLL) were considered the same underlying disease [17].

### EBV serology

The serological analysis was performed at the Department of Pathology, VU medical centre Amsterdam, The Netherlands. Laboratory methods have been described in detail elsewhere [5].

*ELISA:* For the assessment of the general EBV serostatus, we used synthetic peptide-based ELISA assays that measure IgG reactivity to combined immunodominant epitopes of EBNA1 (BKRF1) and VCA-p18 (BFRF3) respectively.

*Immunoblot analysis:* Nuclear extracts of the TPA/butyrate induced HH514.C16 cells were used as source of antigen in immunoblot assays, using standardized procedures as described previously [5]. Distinct antibody diversity patterns revealed by the immunoblot analysis allowed us to categorize subjects according to their EBV overall expression, as defined before [5]. Uncomplicated EBV carriers are characterized by restricted IgG antibody reactivity to a limited number of EBV proteins. Besides EBNA1 and VCA18, the VCA-p40 (BdRF1) and Zebra (BZLF1) proteins are generally recognized by healthy individuals. Patients with infectious mononucleosis (IM) are recognized by a strong response to EAd polypeptides encoded by BMRF1 (EAd-p47/54) and BALF2 (EAd-p138), in the absence of EBNA1 reactivity (IM-pattern). Patients with active EBV infection and ab_EBV activity are characterized by a more diverse pattern of antibody reactivity, yielding a broadly reactive immunoblot profile. For statistical analysis, immunoblot results were grouped into 2 categories, being normal (pattern 1) or abnormal reactive pattern or ab_EBV (pattern 2) (Additional file 1: S1). Patients with an IM-like result were coded as ab_EBV positive, as published before [5]. Individual bands on immunoblot strips were scored independently by 2 individuals according to reactivity from negative to 3+, standardized by reference to a set of control sera analysed in each immunoblot experiment. EBV seropositivity is defined by a positive response in either VCA-p18 and/or EBNA1 IgG ELISA and a positive score in the EBV-immunoblot. Serum samples from cases and controls were tested blind to their disease status.

### Statistical analysis

Odds ratios (OR) were estimated by maximum likelihood using unconditional logistic regression to examine the association between ab_EBV and CLL. Polytomous unconditional logistic regression models were used to compare each CLL stage to controls. All models were adjusted for the frequency matched variables: age (based on tertile distribution of controls: <63, 63-71, 72 or more years), sex and centers (Barcelona, Other). None of the adjustment variables had missing values. Ninety-five percent confidence intervals (CI) for adjusted OR were derived from the variance-covariance matrix of the logistic regression estimators. Potential confounding variables were examined comparing if the unadjusted effect measure differs from the adjusted measure by 10%. The contribution to the models to effect modifications was tested by means of likelihood-ratio tests. A *priori* selected variables included basic socio-economic factors (age at recruitment, region, sex, body mass index coded as normal: 18.0 to 24.9 kg/m^2^, overweight: 25 to 29.9 kg/m^2^ and obese: ≥30 kg/m^2^, education, tobacco consumption, personal history of non-haematological cancer) as well as established risk factor for CLL (family history of haematological cancer) and factors that might be associated with EBV infection (number of siblings coded as 0-1, 2-3 and 4 or more). Alcohol could not be examined due to a high percentage of missing values among controls (12%) and cases (42%). In fully adjusted models, missing values for each adjustment variable were treated as a separate category. A forest plot of the effect modification of smoking on the relation between CLL and ab_EBV was done with black squares indicating OR and vertical lines representing 95% CI. All tests for interactions were made on a multiplicative scale.

Sensitivity analyses were performed comparing patients with different length of time from diagnosis to recruitment (incident versus prevalent) as well as excluding individuals self-reporting gluco-corticosteroid medications. All P-values were two-sided and data analyses were performed using STATA computer software (version 10.1).

## Results

EBV carriership defined by IgG reaction in both the VCA-p18 and EBNA1 ELISA was detected in 98% (N = 302/307) of the 307 individuals included in the analysis. EBV seronegativity was reported for 1 control and 4 cases. Descriptive statistics of the 150 cases (96 CLL Rai 0, 53 CLL Rai I-IV and 1 patient with unknown Rai stage) and 157 controls are shown in Table 1. Most participants were from Barcelona (≈85% of controls) with a mean age for cases and controls of 67 (standard deviation: 10 years) and around 65% of patients with male sex. Overall, there were no statistically significant differences in the distribution between cases and controls with family history of haematological cancer, education, tobacco consumption, body mass index, number of siblings and personal history of other cancer.

**Table 1:** **Socio-demographic and other descriptive characteristics of cases and controls**

	CONTROLS	CASES	CLL Rai0	CLL Rai I-IV
N	157	150 ^b^	96	53
**Region**				
Barcelona	133 (85%)	129 (86%)	86 (90%)	43 (81%)
Other	24 (15%)	21 (14%)	10 (10%)	10 (19%)
*P-value* ^*a*^	*-*	*0.75*	*0.27*	*0.54*
**Age group**				
<63	48 (31%)	44 (29%)	22 (23%)	22 (42%)
63-71	55 (35%)	53 (35%)	38 (40%)	15 (28%)
72+	54 (34%)	53 (35%)	36 (38%)	16 (30%)
mean (SD)	67 (10)	67 (10)	68 (9)	65 (11)
*P-value* ^*a*^	*-*	*0.81*	*0.29*	*0.24*
**Sex**				
Male	102 (65%)	97 (65%)	64 (67%)	32 (60%)
Female	55 (35%)	53 (35%)	32 (33%)	21 (40%)
*P-value* ^*a*^	*-*	*0.96*	*0.78*	*0.55*
**Education**				
Incomplete primary school	49 (31%)	53 (38%)	34 (39%)	19 (37%)
Complete primary school	40 (25%)	47 (33%)	31 (35%)	15 (29%)
Pre-university/technical studies	45 (29%)	25 (18%)	13 (15%)	12 (23%)
University	23 (15%)	16 (11%)	10 (11%)	6 (12%)
*P-value* ^*a*^	*-*	*0.08*	*0.05*	*0.75*
**Tobacco consumption**				
Never	72 (46%)	68 (46%)	42 (45%)	26 (49%)
Former	59 (38%)	61 (41%)	40 (43%)	20 (38%)
Current	25 (16%)	18 (12%)	11 (12%)	7 (13%)
*P-value* ^*a*^	*-*	*0.60*	*0.57*	*0.87*
**Number of siblings**				
0/1	39 (25%)	24 (17%)	14 (16%)	10 (19%)
2 or 3	69 (44%)	57 (41%)	36 (41%)	21 (40%)
4+	49 (31%)	59 (42%)	37 (43%)	21 (40%)
*P-value* ^*a*^	*-*	*0.10*	*0.13*	*0.45*
**Body mass index**				
Normal	51 (34%)	35 (28%)	21 (27%)	13 (28%)
Overweight	77 (52%)	69 (55%)	45 (58%)	24 (51%)
Obese	21 (14%)	22 (17%)	12 (15%)	10 (21%)
*P-value* ^*a*^	*-*	*0.45*	*0.53*	*0.44*
**Personal history of other cancer**				
Never	135 (87%)	119 (84%)	77 (88%)	42 (81%)
Ever	21 (13%)	22 (16%)	11 (13%)	10 (19%)
*P-value* ^*a*^	-	*0.60*	*0.83*	*0.31*
**Family history of haematological cancer**				
No	121 (92%)	119 (89%)	70 (85%)	48 (94%)
Yes	10 (8%)	15 (11%)	12 (15%)	3 (6%)
*P-value* ^*a*^	-	*0.33*	*0.11*	*0.67*

CLL: Chronic lymphocytic leukemia; N: Number; SD: standard deviation.

^a^ *:* All p-values were for heterogeneity except for the age variable with a P-value for trend. ^b^: Rai stage was unknown for one patient.

Numbers do not add up to total number in all instances due to missing value.

As shown in the table of Additional file 1: S2, among controls, the prevalence of ab_EBV patterns decreased with increasing age (P-trend = 0.02). Current smokers were more likely to have aberrant EBV patterns than never or past smokers (P-heterogeneity < 0.001) and no difference was observed in relation to other examined variables. In summary, no confounding variable between the presence of CLL and ab_EBV patterns was identified. Hence, results were further adjusted only for the frequency-matched variables: age, sex and region.

Table 2 shows odds ratio for all cases and by CLL Rai stages in relation to ab_EBV patterns adjusted for age, sex and region. Overall, cases were twice more likely to have ab_EBV patterns than controls (23% of cases versus 11% of controls; OR: 2.44, 95% CI: 1.29 to 4.62; P = 0.006) but when Rai stages were considered, Ab_EBV reactivity was significantly increased only in CLL Rai I-IV, irrespective of treatment status (P-heterogeneity treated versus untreated CLL Rai I-IV: 0.26).

**Table 2:** **Odds ratios of chronic lymphocytic leukemia for aberrant EBV patterns**

	N	N (%) ab_EBV positive	OR ^a^ (95%CI)	***P*** -value
**Controls**	157	17 (11%)	REF	
**Overall**				
All cases	150	34 (23%)	2.44 (1.29 to 4.62)	*0.006*
**By Rai stages**				
CLL Rai 0	96	16 (17%)	1.67 (0.79 to 3.51)	*0.18*
CLL Rai I-IV (untreated^b^)	37	11 (30%)	3.25 (1.35 to 7.82)	*0.008*
CLL Rai I-IV (treated^b^)	16	7 (44%)	7.33 (2.33 to 23.06)	*0.001*

Ab_EBV: aberrant EBV pattern; CLL: Chronic lymphocytic leukemia; CI: confidence interval; N: Number; OR: Odds ratio; REF: reference group. ^*a*^: Logistic regression for overall analysis on cases and controls, multinomial logistic regression otherwise. Odds ratios adjusted for age, sex and region. ^b^: treated for CLL.

**Figure 1:**
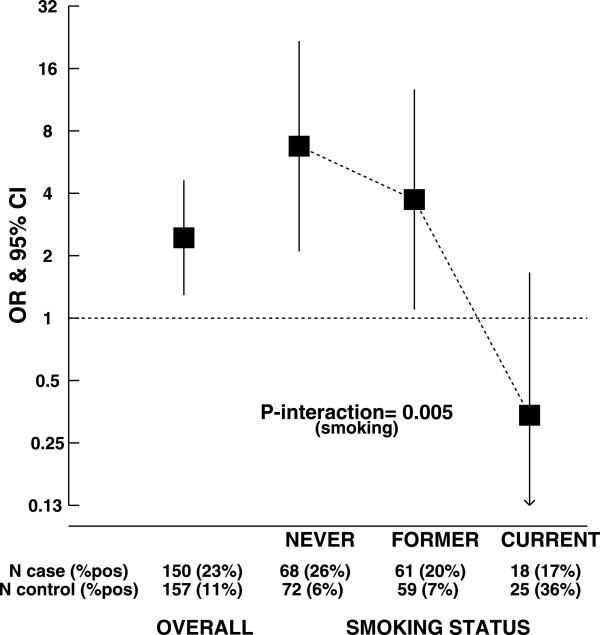
**Association between chronic lymphocytic leukemia and aberrant EBV patterns, by smoking status.** Ab_EBV: aberrant EBV pattern; CLL: Chronic lymphocytic leukemia; CI: confidence interval; OR: Odds ratio; N: Total number; %pos: proportion with aberrant patterns.^a^: Logistic regression adjusted for age, sex and region. ^b^: All tests for interactions were made on a multiplicative scale.

Of the epidemiological characteristics examined (Additional file 1: S2), only smoking status modified the association between ab_EBV and CLL (P-value for interaction between never, former and current smokers: P = 0.005). Overall, a gradual decrease in odds ratios from never, former to current smokers was observed (Figure 1). A strong positive association between ab_EBV and CLL was detected in never smokers (OR for all cases: 6.75, 95% CI: 2.10, 21.71; *P* = 0.001), irrespective of Rai stages and treatment (Additional file 1: S3), whereas, albeit in small numbers, cases being current smokers were 0.3 times less likely to have ab_EBV patterns than controls currently smoking (OR for all cases: 0.34, 95% CI: 0.07, 1.66, *P* = 0.18). A borderline statistical significant interaction with age was found (P = 0.07).

To examine if serological response could be related to the inclusion of prevalent cases with existing condition for a period of time longer than one year, sensitivity analyses were performed but no modification of the findings was observed (Additional file 1: S4). The mean time between diagnosis and recruitment into the study was 2.57 years (standard deviation: 2.97 years and range: 0 year to 16.72 years). The fully adjusted model including the nine variables selected *a priori* gave similar results (OR: 2.54, 95% CI: 1.27 to 5.08; P-value: 0.008). Upon exclusion of individuals self-reporting gluco-corticosteroid medications (N = 5 and N = 8 for controls and cases, respectively) the results were unchanged (data not shown).

## Discussion

Using immunoblot analysis, the present study showed that patients with CLL compared to controls had markedly distinct pattern in EBV-specific polypeptides, in particular clear responses to EA-associated proteins. Although based on small numbers, the association was modified by smoking status with significantly higher ab_EBV-related OR for all CLL stages in never smokers than in current smokers.

Ab_EBV pattern was strongly associated with CLL suggesting an increased viral replication or loss of host control infection [3–5]. Our results were supported by those from the European study EpiLymph that used the same immunoblot technique. [5] In line with our findings, using multiplex technology, previous studies reported an increased risk in patients with higher levels of antibody to EAd [4, 6] but also with EBV DNA loads, both markers of EBV reactive infections [4]. Bertrand *et al* reported non-significant associations with elevated titers against EBV EBNA-2 and VCA, and EBNA1/EBNA-2 ratio less than or equal to 1, but no differences in the titers against EBV-EA prior to diagnosis were observed in 79 CLL compared to those in a set of matched controls [3].

Since CLL cells are generally EBV negative, a direct role of EBV in the aetiology of CLL seems unlikely. We speculate that the increased ab_EBV could also correspond to a response of the B-cell repertoire to replace “sick” lymphocytes (CLL cells) which in turn results with the expansion of latently infected B cells. If further research can confirm this hypothesis, the ab_EBV pattern could then be interpreted as a reactive response that is downplayed among current smokers. Further, ab_EBV reactivation may, in turn, trigger both B- and T-cell activation, which may increase the risk of uncontrolled lymphoproliferations and, hence, higher probability of transforming mutations. Lifetime exposure to infectious agents [18–22] causing a strong and inappropriate immune response or through persistent and chronic antigen stimulation have been suggested to trigger monoclonal B-cell lymphocytosis (MBL), a precursor to CLL and CLL development. EBV could as well be one of the pathogens involved in the indirect deregulation of B-cells. However, as ever in a retrospective study design, a reverse causality bias could not be excluded: the presence of ab_EBV patterns could reflect an existing inefficient immune system of CLL patients.

Of particular interest is the evidence suggesting that the association between ab_EBV patterns and CLL is modified by tobacco smoke. This interaction might provide an explanation for some inconsistencies across publications related to EBV serology. To date and in accordance to our data, large cohort studies [13, 14] and pooled case-control studies [15], comparing the risk of CLL between current and never-smokers have reported no association. EBV infects, persists in human B-cells and reactivates in epithelial cells of the naso- and oro-pharynx during lytic reactivation and transmission via saliva [23]. This direct exposure to tobacco compounds could modulate the life cycle of the virus. The regulation of EBV infection by the host necessitates efficient T-cell–mediated immunity and tobacco smoke has been related to changes in both humoral and cell-mediated immune responses [24]. In particular, chronic inflammation via cytokine production is thought to play an important role in CLL [25] and smoking might affect further the deregulation of inflammatory pathways and T-cell mediated immunity [24] and counterbalance the serological association between EBV and CLL. Furthermore, smoking was recently confirmed as a risk factor for nasopharyngeal carcinoma, a cancer etiologically closely related to aberrant EBV infection in certain populations [12].

Limitations of our study include the small sample size that does not allow detailed examination of the interactions between EBV results and smoking in CLL, overall and by disease severity. The updated guidelines for the classification of CLL and MBL [16, 26] have had a huge impact on the overall classification of CLL, reducing the burden of CLL in about 45% [27, 28]. Unfortunately, CLL Rai 0 could not be disentangled from MBL patients since, for most patients, the absolute B-cell count was unknown at recruitment. Another limitation of the study was the inclusion of prevalent cases [29]. However, incident and prevalent cases did not appear to differ with respect to ab_EBV patterns and the exclusion of prevalent cases did not affect the overall results. Finally, the main advantage of the MCC-Spain study is the recruitment of controls from the general population.

In conclusion, ab_EBV pattern is present in early stages of CLL and is modulated by smoking status. Our data suggest that ab_EBV pattern is likely to reflect a disease response to an existing deficient B-cell pool in CLL rather than a carcinogenic role. Exposure to tobacco smoke should be carefully examined for future studies investigating EBV circulating biomarkers.

## Electronic supplementary material

Additional file 1: **S1** **- Immunoblot EBV reactive patterns.** **S2** - Odds ratios (OR) and 95% confidence interval (CI) for CLL, by various characteristics. **S3** - Odds ratios of CLL by Rai stages for aberrant EBV patterns, stratified by tobacco consumption **S4** - Odds ratios of CLL for aberrant EBV patterns, by different prevalence time periods. (PDF )

## Abbreviations

ab_EBV: Aberrant Esptein-Barr virus
CLL: Chronic lymphocytic leukemia
OR: Odds ratio
CI: Confidence interval.
