# Supplementary material for: Aberrant Epstein-Barr virus antibody patterns and chronic lymphocytic leukemia in a Spanish multicentric case-control study
Source: Infect Agent Cancer. 2015 Feb 9;10:5. doi: 10.1186/1750-9378-10-5 (PMC4429596; doi:10.1186/1750-9378-10-5)
Supplement: Supplementary file 1 — Additional file 1: S1 - Immunoblot EBV reactive patterns. S2 - Odds ratios (OR) and 95% confidence interval (CI) for CLL, by various characteristics. S3 - Odds ratios of CLL by Rai stages for aberrant EBV patterns, stratified by tobacco consumption S4 - Odds ratios of CLL for aberrant EBV patterns, by different prevalence time periods. (PDF ) [file 13027_2014_525_MOESM1_ESM.pdf]

Supplementary information

S1 - Immunoblot EBV reactive patterns

S2 - Odds ratios (OR) and 95% confidence interval (CI) for CLL, by various characteristics

S3 - Odds ratios of CLL by Rai stages for aberrant EBV patterns, stratified by tobacco consumption

S4 - Odds ratios of CLL for aberrant EBV patterns, by different prevalence time periods

S1 – - Immunoblot EBV reactive patterns

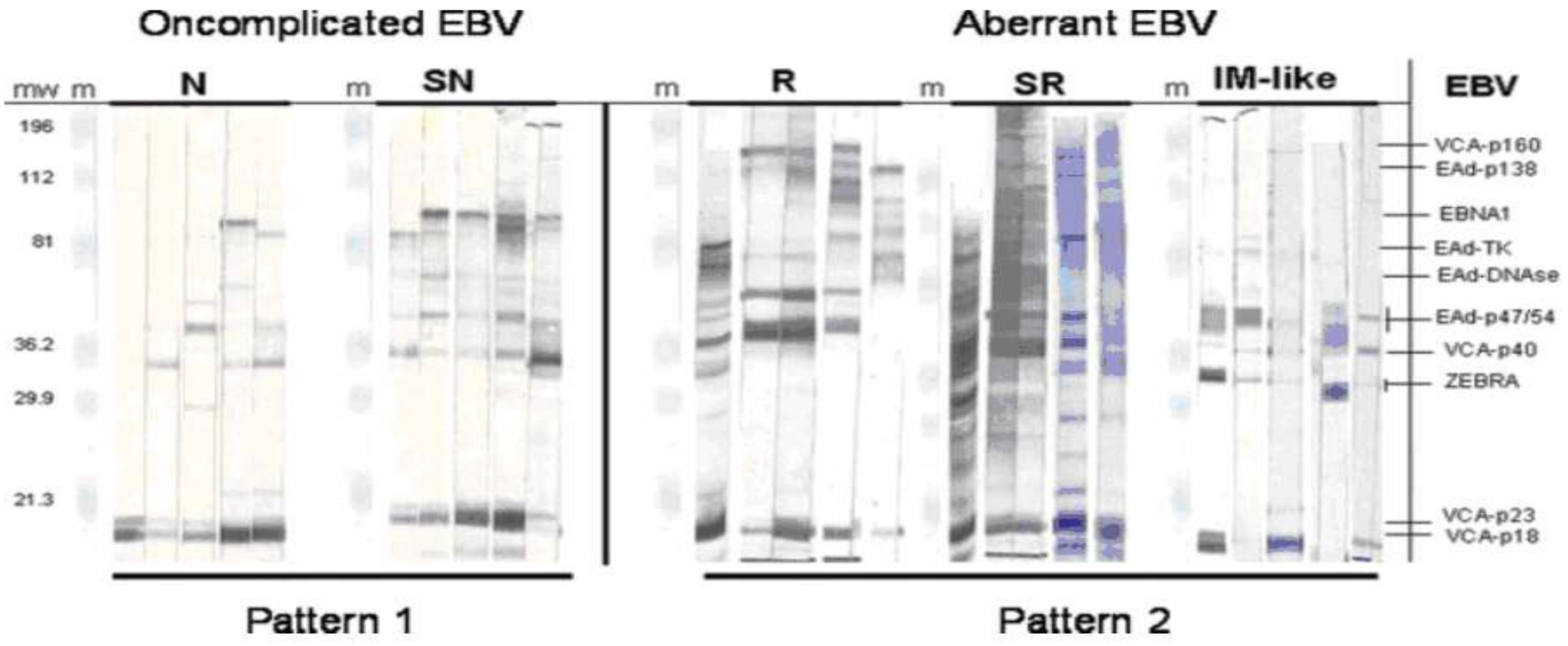

Immunoblot analysis reveals the underlying of antibody responses to individual EBV proteins as detailed by Middeldorp et al. [1–6] In the data analysis, N (normal) and SN (strong normal) are considered as normal pattern and is used as a reference pattern-1. R (reactive), SR (strong reactive) and IM-like, all including clear responses to EA-associated proteins, are grouped together and refer in the text as ab_EBV. The IM-like pattern lacks EBNA1 reactivity in the presence of EA and VCA reactivity. [2,3]

S2 - Odds ratios (OR) and 95% confidence interval (CI) for CLL, by various characteristics

	N Controls/cases	CONTROLS ab_EBV positive N(%)	CLL ab_EBV positive N(%)	OR[a] & 95% CI	*P*-value	*P*-value for interaction [b]
**All cases and controls**	**157/150**	**17 (11%)**	**34 (23%)**	**2.44 (1.29 to 4.62)**	***0.006***	
**Region**						
Barcelona	133/129	14 (11%)	30 (23%)	2.59 (1.30 to 5.15)	*0.007*	
Other	24/21	3 (13%)	4 (19%)	1.89 (0.31 to 11.56)	*0.49*	0.65
		P-value [c] =0.78				
**Age group**						
<63	48/44	9 (19%)	9 (20%)	1.16 (0.41 to 3.30)	*0.79*	
63-71	55/53	6 (11%)	12 (23%)	2.33 (0.80 to 6.81)	*0.12*	
72+	54/53	2 (4%)	13 (25%)	8.64 (1.83 to 40.67)	*0.006*	0.07
		P-value [c] = 0.02				
**Sex**						
Male	102/97	11 (11%)	18 (19%)	1.87 (0.83 to 4.22)	*0.13*	
Female	55/53	6 (11%)	16 (30%)	3.62 (1.27 to 10.30)	*0.02*	0.33
		P-value [c] = 0.98				
**Education**						
Lower education	89/100	11 (12%)	24 (24%)	2.25 (1.03 to 4.93)	*0.04*	
Higher education	68/41	6 (9%)	8 (20%)	2.30 (0.71 to 7.47)	*0.16*	0.92
		P-value [c] = 0.37				
**Tobacco consumption**						
Never	72/68	4 (6%)	18 (26%)	6.75 (2.10 to 21.71)	*0.001*	
Former	59/61	4 (7%)	12 (20%)	3.73 (1.10 to 12.68)	*0.04*	
Current	25/18	9 (36%)	3 (17%)	0.34 (0.07 to 1.66)	*0.18*	0.005
		P-value [c] < 0.001				
**Number of siblings**						
0/1	39/24	5 (13%)	7 (29%)	2.54 (0.63 to 10.18)	*0.19*	
2 or 3	69/57	5 (7%)	8 (14%)	1.94 (0.59 to 6.41)	*0.28*	
4+	49/59	7 (14%)	17 (29%)	2.40 (0.89 to 6.51)	*0.09*	0.95
		P-value [c] = 0.43				
**Body mass index**						
normal	51/35	6 (12%)	12 (34%)	4.42 (1.41 to 13.84)	*0.01*	
overweight	77/69	8 (10%)	10 (14%)	1.45 (0.53 to 3.94)	*0.47*	
obese	21/22	3 (14%)	6 (27%)	2.69 (0.52 to 13.99)	*0.24*	0.43
		P-value [c] = 0.88				
**Personal history of other cancer**						
Never	135/119	16 (12%)	26 (22%)	2.13 (1.08 to 4.23)	*0.03*	
Ever	21/22	1 (5%)	6 (27%)	11.93 (1.14 to 124.43)	*0.04*	0.23
		P-value [c] = 0.33				
**Family history of haematological cancer**						
No	147/125	17 (12%)	28 (22%)	2.25 (1.16 to 4.35)	*0.02*	
Yes	10/15	0 (0%)	4 (27%)	NE		0.12
		P-value [c] = 0.26				

Ab_EBV: aberrant EBV pattern; CLL: Chronic lymphocytic leukemia; CI: confidence interval; N: Number; OR: Odds ratio.
[a]: Logistic regression adjusted as appropriate for age, sex and region. [b]: All tests for interactions were made on a multiplicative scale. [c]: All p-values were for heterogeneity except for the age variable with a P-value for trend. Numbers do not add up to total number in all instances due to missing value.

S3 - Odds ratios of CLL by Rai stages for aberrant EBV patterns, stratified by tobacco consumption

	NEVER SMOKER				FORMER SMOKER				CURRENT SMOKER			
	**N**	**N (%) ab_EBV positive**	**OR ^{a} (95%CI)**	***P*-value**	**N**	**N (%) ab_EBV positive**	**OR ^{a} (95%CI)**	***P*-value**	**N**	**N (%) ab_EBV positive**	**OR ^{a} (95%CI)**	***P*-value**
Controls	72	4 (6%)	REF		59	4 (7%)	REF		25	9 (36%)	REF	
**Overall**												
All cases	68	18 (26%)	6.75 (2.10 to 21.71)	*0.001*	61	12 (20%)	3.73 (1.10 to 12.68)	*0.04*	18	3 (17%)	0.34 (0.07 to 1.66)	*0.18*
**By Rai stages**												
CLL Rai 0	42	9 (21%)	5.32 (1.47 to 19.32)	*0.01*	40	5 (13%)	2.33 (0.56 to 9.66)	*0.25*	11	1 (9%)	0.21 (0.02 to 2.28)	*0.20*
CLL Rai I-IV (untreated[b])	18	6 (33%)	8.81 (2.07 to 37.51)	*0.003*	12	3 (25%)	3.77 (0.64 to 22.25)	*0.14*	7	2 (29%)	0.56 (0.08 to 4.03)	*0.56*
CLL Rai I-IV (treated[b])	8	3 (38%)	9.53 (1.47 to 61.91)	*0.02*	8	4 (50%)	17.36 (2.84 to 106.17)	*0.002*	0	0	-	

Ab_EBV: aberrant EBV pattern; CLL: Chronic lymphocytic leukemia; CI: confidence interval; N: Number; OR: Odds ratio. [a]: Logistic regression for overall analysis on cases and controls, multinomial logistic regression otherwise. Odds ratios adjusted for age, sex and region. [b]: treated for CLL.

S4 - Odds ratios of CLL for aberrant EBV patterns, by different prevalence time periods

						**Restricted to never smokers**			
		**N**	**N (%) ab_EBV positive**	**OR[a] (95%CI)**	***P*-value**	**N**	**N (%) ab_EBV positive**	**OR[a] (95%CI)**	***P*-value**
All cases	Controls	157	17 (11%)	REF		72	4 (6%)	REF	
	CLL	150	34 (23%)	2.44 (1.29 to 4.62)	*0.006*	68	18 (26%)	6.75 (2.10 to 21.71)	*0.001*
<u>Cases with time from diagnosis to interview:</u>[b]									
Incident (≤ 1 year)									
	CLL	44	11 (25%)	2.80 (1.18 to 6.63)	*0.02*	18	6 (33%)	9.14 (2.11 to 39.51)	*0.003*
Prevalent (> 1 year)									
	CLL	105	23 (22%)	2.39 (1.20 to 4.78)	*0.01*	50	12 (24%)	6.66 (1.88 to 23.57)	*0.003*

Ab_EBV: aberrant EBV pattern; CLL: Chronic lymphocytic leukemia; N: Number; OR: Odds ratio; CI: confidence interval.
[a]: Logistic regression for overall analysis on cases and controls. Odds ratios adjusted for age, sex and region.
[b]: P-heterogeneity for the case-case analysis: 0.57 (overall) and 0.45 (in never smokers)

REFERENCES

1. De Sanjosé S, Bosch R, Schouten T, Verkuijlen S, Nieters A, Foretova L, et al. Epstein-Barr virus infection and risk of lymphoma: immunoblot analysis of antibody responses against EBV-related proteins in a large series of lymphoma subjects and matched controls. Int J Cancer [Internet]. 2007 Oct 15 [cited 2014 Jan 8];121(8):1806–12. Available from: http://www.ncbi.nlm.nih.gov/pubmed/17557295

2. Middeldorp JM, Herbrink P. Epstein-Barr virus specific marker molecules for early diagnosis of infectious mononucleosis. J Virol Methods [Internet]. 1988 Sep [cited 2014 Feb 6];21(1-4):133–46. Available from: http://www.ncbi.nlm.nih.gov/pubmed/2846609

3. Van Grunsven WM, Nabbe A, Middeldorp JM. Identification and molecular characterization of two diagnostically relevant marker proteins of the Epstein-Barr virus capsid antigen complex. J Med Virol [Internet]. 1993 Jun [cited 2014 Feb 6];40(2):161–9. Available from: http://www.ncbi.nlm.nih.gov/pubmed/8395556

4. Meij P, Vervoort MB, Aarbiou J, van Dissel P, Brink A, Bloemena E, et al. Restricted low-level human antibody responses against Epstein-Barr virus (EBV)-encoded latent membrane protein 1 in a subgroup of patients with EBV-associated diseases. J Infect Dis [Internet]. 1999 May [cited 2014 Feb 6];179(5):1108–15. Available from: http://www.ncbi.nlm.nih.gov/pubmed/10191211

5. Meij P, Vervoort MBHJ, Bloemena E, Schouten TE, Schwartz C, Grufferman S, et al. Antibody responses to Epstein-Barr virus-encoded latent membrane protein-1 (LMP1) and expression of LMP1 in juvenile Hodgkin’s disease. J Med Virol [Internet]. 2002 Nov [cited 2014 Feb 6];68(3):370–7. Available from: http://www.ncbi.nlm.nih.gov/pubmed/12226824

6. Fachiroh J, Schouten T, Hariwiyanto B, Paramita DK, Harijadi A, Haryana SM, et al. Molecular diversity of Epstein-Barr virus IgG and IgA antibody responses in nasopharyngeal carcinoma: a comparison of Indonesian, Chinese, and European subjects. J Infect Dis [Internet]. 2004 Jul 1;190(1):53–62. Available from: http://www.ncbi.nlm.nih.gov/pubmed/15195243
